# Comparison of long-term effects of metformin on longevity between people with type 2 diabetes and matched non-diabetic controls

**DOI:** 10.1186/s12889-023-15764-y

**Published:** 2023-05-02

**Authors:** Joshua Stevenson-Hoare, Ganna Leonenko, Valentina Escott-Price

**Affiliations:** 1 MRC Centre for Neuropsychiatric Medicine and Clinical Neuroscience, Cardiff University, Hadyn Ellis Building, Maindy Road, Cardiff, CF24 4HQ UK; 2 Dementia Research Institute, Cardiff University, Cardiff, UK

**Keywords:** Diabetes, Metformin, Sulphonylurea, Longevity, Survival

## Abstract

**Background:**

Metformin, a medication for type 2 diabetes, has been linked to many non-diabetes health benefits including increasing healthy lifespan. Previous work has only examined the benefits of metformin over periods of less than ten years, which may not be long enough to capture the true effect of this medication on longevity.

**Methods:**

We searched medical records for Wales, UK, using the Secure Anonymised Information Linkage dataset for type 2 diabetes patients treated with metformin (N = 129,140) and sulphonylurea (N = 68,563). Non-diabetic controls were matched on sex, age, smoking, and history of cancer and cardiovascular disease. Survival analysis was performed to examine survival time after first treatment, using a range of simulated study periods.

**Findings:**

Using the full twenty-year period, we found that type 2 diabetes patients treated with metformin had shorter survival time than matched controls, as did sulphonylurea patients. Metformin patients had better survival than sulphonylurea patients, controlling for age. Within the first three years, metformin therapy showed a benefit over matched controls, but this reversed after five years of treatment.

**Interpretation:**

While metformin does appear to confer benefits to longevity in the short term, these initial benefits are outweighed by the effects of type 2 diabetes when patients are observed over a period of up to twenty years. Longer study periods are therefore recommended for studying longevity and healthy lifespan.

**Evidence before this study:**

Work examining the non-diabetes outcomes of metformin therapy has suggested that there metformin has a beneficial effect on longevity and healthy lifespan. Both clinical trials and observational studies broadly support this hypothesis, but tend to be limited in the length of time over which they can study patients or participants.

**Added value of this study:**

By using medical records we are able to study individuals with Type 2 diabetes over a period of two decades. We are also able to account for the effects of cancer, cardiovascular disease, hypertension, deprivation, and smoking on longevity and survival time following treatment.

**Implications of all the available evidence:**

We confirm that there is an initial benefit to longevity of metformin therapy, but this benefit does not outweigh the negative effect on longevity of diabetes. Therefore, we suggest that longer study periods are required for inference to be made about longevity in future research.

## Introduction

Metformin is a first-line medication used for the treatment of Type II diabetes (T2D) [1]. In addition to its primary purpose in diabetes treatment, metformin has been heavily associated with benefits to both total lifespan, and healthy lifespan [2, 3]. Interventional and observational research has suggested benefits for cognitive decline [4, 5], reduced rates of cancer [6–8], reduced incidence of diabetes in individuals with elevated blood glucose levels [9], and reduced cardiovascular risk factors [10–12], among others. Metformin is also prescribed for some other conditions such as polycystic ovary syndrome, where it has been demonstrated to aid in fertility treatments [4]. The associated benefits of metformin are particularly evident when it is compared to other treatments, such as sulphonylurea therapy or thiazolidinediones, which has not been shown to have the same benefits [13, 14].

However, observational studies of metformin and longevity rarely examine individuals for periods longer than a few years. For example, Bannister et al. [14] observed a benefit to longevity for individuals treated with metformin over a five-year period, as did Cheng et al. [13]. Landman et al. [15] found reduced cancer mortality in metformin-treated participants followed-up after 9·6 years, which was sufficient for cancer mortality, but may not be long enough for all-cause mortality interpretation. However, Tinetti et al. [16] followed individuals for between four and six years, and did not find a benefit to longevity of metformin treatment. The UK Prospective Diabetes Study (UKPDS) [17] has been used to study long-term effects (greater than twenty years) of longevity related to diabetes therapies and has been used to find a benefit of metformin for diabetes-related mortality causes [18]. However, this cohort only contained individuals either with diabetes or at high-risk, so is not informative about longevity relative to the general population.

Evidence from animal studies has been largely positive in suggesting a benefit of metformin [19–22], but by the nature of this research it is limited in the timespan available for study. Although, even here, evidence appears to be species-dependent with Drosophila showing reduced lifespan when supplemented with metformin [23, 24], and no effect on longevity in rats [25].

As longevity effects only become clear over whole lifespans, longer study periods are more powerful to detect real effects. Whereas cohort studies need to acquire participants through recruitment or surveying, use of archived medical records permits access to a far greater participant pool for a lower time and cost investment. Hence, it is possible to examine the course of a person’s life from treatment time until death, and thereby get a more representative account of their longevity.

Our primary aim was to study longevity in diabetes patients treated with metformin therapy over a period of two decades. As there is some evidence that late-life diabetes can be protective against neurodegenerative disorders [26] (as is higher body-mass index [27] which is associated with T2D), as a secondary aim, we examined survival times for individuals treated with metformin and sulphonylurea therapy who were elderly (over 70) at the time of first diabetes treatment.

For this we used the Secure Anonymised Information Linkage (SAIL) database, an archive of medical and demographic records for the Welsh population in the UK. We extracted information for all T2D patients treated with metformin and sulphonylurea therapy and, using a matched controls design, analysed survival time following first treatment.

## Methods

### Datasets and data extraction

The SAIL database is a virtual platform that provides secure anonymised linkage between medical datasets using anonymised identification codes. All records in SAIL are from the population of Wales, UK. For this research we used the Welsh Longitudinal General Practitioner (WLGP) and Welsh Demographic Services (WDSD) datasets. The WLGP dataset contains records from primary care physicians, including symptoms, diagnoses, medication prescriptions, and referrals. The WDSD dataset contains demographic records including date of birth (DOB), date of death (DOD), sex, and address (Welsh or non-Welsh).

To extract T2D patients, we used NHS Read codes (CV2, CV3) to identify all individuals with an active diagnosis of T2D between January 1st 1999 and December 31st 2018, a period of twenty years. To be considered an active diagnosis, individuals had to have been prescribed diabetes medication on at least two instances a minimum of 180 days apart. For medications, we extracted prescriptions of metformin, sulphonylurea, and thiazolidinedione and matched these to T2D patients. Potential controls were all individuals who were not diagnosed with any form of diabetes. We also extracted information from WLGP for all individuals on smoking status, cancer, cardiovascular disease including heart failure (CVD), hypertensive disease, and deprivation index (Welsh Index of Multiple Deprivation [28]) to account for these factors in our analyses.

### Analysis

Survival analysis was performed using an accelerated failure time model (AFT) using a log-logistic distribution, as the data failed the proportional hazards assumption (as hazard was not constant over time with different predictor and covariate levels) required for a Cox regression. This was performed using the “eha” package in R [29]. All models were adjusted for age at first treatment.

In “cases” survival time was calculated as the number of days from start of therapy until death or censorship, minus 180 days. This value was used as it represents approximately six months of treatment, which should be sufficient to allow the effect of the medication to begin [14]. Patients in both treatment groups (metformin and sulphonylurea) were matched one-to-one to non-diabetic controls using sex, date of birth (± 1 year), history of cancer, history of CVD, hypertension (excluding hypertension related to pregnancy), deprivation index, and smoking status. The scripts used to match these individuals were adapted from work published by the UK National Health Service (NHS) R Community [30].

For controls, survival time was started from the date of first therapy of their matched case, minus 180 days. Censorship occurred for individuals who were not listed as dead by December 31st 2018. We also extended the analysis by truncating our data to a variable number of years following first therapy and assigning censorship at that amount of time following the start date, to simulate shorter study periods.

Survival time ratios (STR) were calculated as the exponent of the beta coefficient from the survival model. Log-rank tests were used to assess differences between groups.

To address a possibility of a positive survival advantage of controls who never develop diabetes, we ran a Cox-regression in the whole population at the beginning of the study (1999). We examined survival rate comparing T2D people on metformin (coded as 1) and people who did not have T2D in 1999 but might have diabetes later in life (coded as 0), adjusting for age in 1999, sex, smoking status, cancer, CVD, hypertensive disease and deprivation index. People who had T2D who were treated with sulphonylurea were excluded from the control group. A separate model was run for T2D people on sulphonylurea, excluding individuals treated with metformin. Hazard Ratios were calculated as the exponent of the beta coefficient from the Cox-regression model.

## Results

We extracted 129,140 T2D patients treated with metformin and 68,108 T2D patients treated with sulphonylurea. We also extracted 1,274 T2D patients treated with thiazolidinedione, however there were not enough individuals per year to obtain adequate power for further analysis so these were dropped. An overview of included individuals is shown in Table 1. There was no difference in sex balance between these two treatment groups (*p* = 0·705), or smoking rate (*p* = 0·085). However, sulphonylurea therapy patients were older than metformin patients at first treatment, (*p* < 1 × 10^− 50^).

Within sulphonylurea therapy patients, 55·8% (N = 38,026) had been treated with metformin. Conversely, only 11·1% (N = 14,357) of metformin therapy patients had been treated with sulphonylurea. We examined whether there were overlaps in the prescription of these medications, and found that 94·0% of patients with prescription history of both treatments had an overlap between treatments. This is in line with NHS guidance [31], which recommends maintenance of the primary T2D treatment alongside additional treatments. Indeed, the median duration of treatment overlap was 7·63 years [IQR = 7.40].

To ensure that individuals in our dataset were regular users of each medication, we looked at the frequency of medication events (i.e., prescriptions) for each individual. Frequency of prescription was highly correlated with time since first prescription in metformin (Pearson’s *r* = 0·740, *p* < 1 × 10^− 99^) and in sulphonylurea (Pearson’s *r* = 0·759, *p* < 1 × 10^− 99^). The median ratio of days since first prescription to frequency of prescription was 30·1 for metformin and 28.5 for sulphonylurea. This represents approximately one prescription per month in both cases, confirming that we have indeed captured regular medication users.

**Table 1:** Demographic overview of metformin and sulphonylurea therapy T2D patients

	Metformin patients	Sulphonylurea patients
N individuals	129,140	68,108
% males / % females	56·1 / 43·9	56·3 / 43·7
Age at first treatment (SD)	59·0 (12·8)	61·1 (12.5)
Cancer	18·7%	21·2%
Cardiovascular disease	87·2%	88·7%
Smokes	51·4%	51·6%
Hypertensive disease	65·0%	67·2%

Adjusting for age difference, sulphonylurea therapy patients were more likely to have had cancer (OR = 1·011, *p* = 2·16 × 10^− 10^) at any point, more likely to have had CVD at any point (OR = 1·006, *p* = 3·02 × 10^− 5^), and more likely to be hypertensive (OR = 1·008, *p* = 1.78 × 10^− 4^) than metformin therapy patients.

When we excluded individuals who had cancer or CVD prior to first treatment for T2D diabetes (N = 15,488, N = 144,615, for cancer and CVD, respectively), patients treated with sulphonylurea were still more likely to have had cancer than patients treated with metformin (OR = 1·12, *p* = 1·53 × 10^− 14^), accounting for age. Sulphonylurea therapy patients were also more likely to experience CVD following treatment than metformin patients (OR = 1·17, *p* = 2·94 × 10^− 17^), accounting for age.

We found that both metformin therapy patients and sulphonylurea therapy patients had shorter survival times than non-diabetic controls, STR = 0·819 (*p* < 1 × 10^− 50^) and STR = 0·778 (*p* < 1 × 10^− 50^), respectively. When we excluded sulphonylurea therapy patients who were also being treated with metformin, the remaining patients had even shorter survival times than non-diabetic controls, STR = 0·693 (*p* < 1 × 10^− 50^), which was significantly lower than the whole sulphonylurea group (p < 0.05). For completeness, we also looked at just those sulphonylurea therapy patients who were also being treated with metformin. Compared to non-diabetic controls, these individuals still had shorter survival, STR = 0·799 (*p* < 1 × 10^− 50^), but this STR was greater than that found using whole sulphonylurea group (p < 0.05). These results were still significantly different to the metformin vs. controls model (p < 0.05).

Similar observations for diabetes treatments were obtained when we ran a Cox regression including all living people with/without diabetes in year 1999 for metformin (hazard ratio HR = 1·69, *p* = 6·72 × 10^− 23^) and sulphonylurea (HR = 1·59, *p* = 2·92 × 10^− 29^). Note that a higher hazard ratio indicates greater likelihood of death, equivalent to a lower STR. However, as this is a Cox regression rather than an accelerated failure time (AFT) model, direct comparison between the strength of effects cannot be made.

Examining survival times for individuals who were elderly (over 70) at the time of first diabetes treatment, metformin patients still had lower survival time than non-diabetic matched controls, STR = 0·915 (*p* = 1·11 × 10^− 16^), as did sulphonylurea therapy patients, STR = 0·858 (*p* < 1 × 10^− 50^).

**Table 2:** Survival model results per simulated study period length, for diabetes therapy patients versus matched controls

	Metformin patients		Sulphonylurea patients	
Truncated length	STR	log-rank *p*	STR	log-rank *p*
1 year	1·237	1·06 × 10^− 10^	0·991	0·868
2 years	1·098	2.57 × 10^− 5^	0·927	0·008
3 years	1·014	0·435	0·886	3·33 × 10^− 8^
4 years	0·972	0·049	0·875	2·02 × 10^− 13^
5 years	0·943	3·78 × 10^− 6^	0·862	< 1 × 10^− 50^
6 years	0·913	1·44 × 10^− 15^	0·844	< 1 × 10^− 50^
7 years	0·894	< 1 × 10^− 50^	0·827	< 1 × 10^− 50^
8 years	0·879	< 1 × 10^− 50^	0·815	< 1 × 10^− 50^
···	···	···	···	···
20 years	0·821	< 1 × 10^− 50^	0·778	< 1 × 10^− 50^

As our data covered a period of twenty years, we were able to investigate survival times for shorter periods. The results revealed that T2D patients treated with metformin had significantly greater survival times than non-diabetic controls in the first year (STR = 1·237, *p* = 1·06 × 10^− 10^). However, this became non-significant after three years, and reversed after four years (STR = 0·972, *p* = 0·049), with survival time ratio decreasing further with longer study periods. Sulphonylurea patients had shorter survival times than non-diabetic controls at all study periods, but this difference was only significant for periods of two years (STR = 0·927, *p* = 0·008) or longer. The results of these survival models for both therapy treatments are shown in Table 2 for one to eight years, and for the full twenty year period. Between eight and twenty years STR decreased for both metformin and sulphonylurea comparisons, and so are not presented for simplicity.

## Discussion

We examined longevity in T2D patients treated with metformin therapy and compared them to matched controls and T2D patients treated with sulphonylurea therapy. Using archival medical records from the SAIL databank provided us with a larger participant pool and longer study period than is available from most observational methods. Metformin therapy patients also had better survival than sulphonylurea therapy patients for all investigated study periods following treatment start.

Looking at individuals over a period of up to twenty years we showed that T2D patients had shorter survival times after first treatment than matched controls. When the study period was artificially truncated, we found a statistically significant benefit of metformin therapy for longevity over matched non-diabetic controls within the first three years. However, this benefit disappeared when we looked over longer periods of time (after five years). This suggests that benefits of metformin may be short-term only and/or the longer-term benefits of metformin are negated by the life-shortening effects of T2D and associated comorbidities. An alternative explanation is that T2D patients experience better short-term survival outcomes following treatment due to lifestyle adjustment (as recommended by doctors) [32]. However, we did not see a benefit to longevity in the short-term for sulphonylurea therapy patients who would presumably be motivated to improve their lifestyle in the same way.

Metformin has been linked to lower mortality due to cancer [15], and to reduced CVD risk [10–12]. Compared to the sulphonylurea therapy group, we did see significantly lower lifetime prevalence of cancer, and lower rates of cardiovascular disease. Excluding individuals with history of cancer and CVD prior to first treatment, these differences were even larger. This finding is supportive of the protective effects of metformin for cancer and CVD compared to other diabetes treatments. However, as we used non-diabetic controls who were matched on cancer and CVD status to the diabetic cases, we are unable to distinguish if there is a benefit of this treatment.

A limitation of this study is that metformin is a mainline medication that is commonly prescribed as a first treatment for T2D^1^, whereas sulphonylurea therapy is less commonly prescribed [33]. As a result, there may be systematic differences between these groups that are not detected from cancer, CVD, and smoking rates. We did see a difference in age, with sulphonylurea therapy patients being older than metformin therapy patients. Furthermore, 55·5% of sulphonylurea therapy patients had been treated with metformin, whereas only 11·1% of metformin therapy patients had been treated with sulphonylurea.

Further examination showed that the vast majority of individuals with a history of both medications in fact had an overlap between these treatments. As we discuss above, NHS guidance is for the maintenance of the primary treatment in addition to further treatments. While this large overlap is representative of the reality of T2D treatment, which is highly dependent on individual response, this may cause longevity differences between treatments to reflect differences in disease severity, comorbidities, or diabetes response rather than effects of the medications themselves. Excluding patients who were treated with metformin concurrently with sulphonylurea worsened the survival time ratio (STR) for sulphonylurea, and only looking at patients on both therapies improved the STR. As the median time difference between starting metformin and starting sulphonylurea was more than seven years, this suggests that metformin remains beneficial for survival with long-term usage, although even with multiple medications it cannot overcome the deleterious effects of T2D on longevity.

Two important factors in T2D are body-mass index (BMI) and the haemoglobin A1c (HbA1c) test. While these data are available for some individuals in the SAIL databank, they are not found universally. For example, BMI is only available for approximately 15% of individuals at any timepoint, and is not necessarily available for the start of our study period. Although HbA1c is more widely available for individuals with diabetes than BMI, there are very few non-diabetic controls with this information. As these are variables that change over time it is not possible to include them in a survival analysis, even if they were more widely available. Consequently, we did not account for the effects of BMI and HbA1c.

In conclusion, previous work using periods less than ten years have shown either positive effects of metformin on T2D survival time [13, 14], or non-significant effects [16]. By using medical records which cover a period of two decades, we have greater power and can examine the effects of diabetes treatment in the long-term. Our research suggests that any short-term benefits of metformin are outweighed by the negative effects of T2D over long periods of time, although we replicated that there may be a benefit of metformin over sulphonylurea therapy. We demonstrate that for examining longevity, archival medical records should be employed wherever possible to study the effects of medication in the long term.

## Data Availability

The datasets supporting the conclusion of this research are accessible via the SAIL platform. Application to view and use this data must be approved by the Information Governance Review Panel (IGRP). For more information see the SAIL guidelines at https://saildatabank.com/data/. No new data were created as part of this study.
